# Using Complex Network Analysis for Optimization of Water Distribution Networks

**DOI:** 10.1029/2020WR027929

**Published:** 2020-08-18

**Authors:** Robert Sitzenfrei, Qi Wang, Zoran Kapelan, Dragan Savić

**Affiliations:** ^1^ Unit of Environmental Engineering University of Innsbruck Innsbruck Austria; ^2^ School of Civil and Transportation Engineering Guangdong University of Technology Guangzhou China; ^3^ Faculty of Civil Engineering and Geosciences, Department of Water Management Delft University of Technology Delft Netherlands; ^4^ Centre for Water Systems University of Exeter Exeter UK; ^5^ KWR Water Cycle Research Institute Nieuwegein Netherlands

**Keywords:** graph analysis, edge betweenness centrality, large networks, characteristics of optimal WDSs

## Abstract

The optimization of water networks supports the decision‐making process by identifying the optimal trade‐off between costs and performance (e.g., resilience and leakage). A major challenge in the domain of water distribution systems (WDSs) is the network (re)design. While the complex nature of WDS has already been explored with complex network analysis (CNA), literature is still lacking a CNA of optimal water networks. Based on a systematic CNA of Pareto‐optimal solutions of different WDSs, several graph characteristics are identified, and a newly developed CNA design approach for WDSs is proposed. The results show that obtained designs are comparable with results found by evolutionary optimization, but the CNA approach is applicable for large networks (e.g., 150,000 pipes) with a substantially reduced computational effort (runtime reduction up to 5 orders of magnitude).

## Key Points

- Patterns of optimal water networks are identified with complex network analysis
- A network theory‐based design approach is developed, which partly outperforms a multiobjective evolutionary design approach
- The new approach achieves runtime reductions up to 5 orders of magnitude and is capable of designing large networks (>150,000 pipes)

## 1 Introduction

Water networks are an essential part of urban infrastructure, and major investments are associated with providing the required capacity (Cunha & Marques, 2020; Johns et al., 2019; Wang et al., 2017).

The optimization of the water networks supports the decision‐making process by ensuring that with a given investment, the maximum gain (e.g., performance, resilience, and robustness) can be achieved. Evolutionary algorithms are widely used in literature to optimize environmental problem formulations and especially for such water networks (Maier et al., 2019). For water distribution systems (WDSs), many different aspects have been the focus of optimization (Mala‐Jetmarova et al., 2017). A major challenge is the design of gravity‐driven pipe networks (De Corte & Sörensen, 2013), which can be addressed by employing multiobjective evolutionary algorithms (Wang et al., 2017). Such procedures result in a set of trade‐off solutions (known as the Pareto front), which can be the basis for decision making. While the process of obtaining these different solutions is explored extensively, the graph characteristics of these optimal networks are hardly explored.

The optimal design of WDSs is an nondeterministic polynomial (NP) time hard problem, which cannot be solved in polynomial time. For large networks, this is computationally intensive, as the number of possible solutions grows exponentially. Therefore, the applicability of evolutionary algorithms is limited to networks with a few thousand pipes, which is a limitation; nowadays, all‐mains models can have hundreds of thousands of pipes. WDSs are complex networks and are composed of multiple interconnected elements, which interact in a complex, nontrivial way. Nevertheless, the strength of complex network analysis (CNA) is in providing a better understanding of the characteristics of WDSs, and it is especially capable of investigating extremely large networks, that is, with millions of edges (Csardi & Nepusz, 2006). Therefore, CNA could improve the applicability of (evolutionary) optimization procedures for extremely large, real‐world WDSs.

CNA has gained more attention in WDS analysis because basic structures and in‐depth understanding can be obtained about the network being analyzed. Yazdani and Jeffrey (2011) explored CNA for practical applications in WDS analysis such as the interplay between network structure and operational efficiency, reliability, and robustness. Yazdani and Jeffrey (2012a) applied network theory to quantify the structural robustness of water distribution networks (WDNs), and they performed vulnerability analysis with weighted, directed graphs of WDS (Yazdani & Jeffrey, 2012b). Ulusoy et al. (2018) investigated the potential of graph metrics as surrogate network measures for resilience analysis. They found that WDSs cannot be sufficiently characterized by graph metrics based on unweighted graphs. Pagano et al. (2019) compared graph‐based to global resilience approaches. Meng et al. (2018) developed a framework for mapping resilience performance and network topological attributes for WDSs and outlined the importance of a specific WDS feature: the locations of sources in WDSs. Torres et al. (2017) explored the topological effects in WDSs using graph theory and showed strong correlations between graph theory metrics and WDS performance measures. Zischg, Klinkhamer, et al. (2019) used complex network theory to describe the temporal evolution of both water supply and drainage infrastructures. They showed that nodal degree distributions in a dual representation of the temporal transition of a water network have almost constant slopes on log‐log plots and can be described with a truncated power law distribution.

Graph‐based methods were also successfully applied for automated creation of district metered areas in WDSs (Di Nardo & Di Natale, 2011; Diao et al., 2013), determining reliability in the context of the isolation valve system (Giustolisi, 2020; Zischg, Reyes‐Silva, et al., 2019), for topological clustering (Perelman & Ostfeld, 2011), or domain analysis (Simone et al., 2019). For classification of WDS, graph theory‐based metrics are used (Hwang & Lansey, 2017) also in order to characterize features like vulnerability (Giustolisi et al., 2017) or to obtain useful information about emerging hydraulic behavior of WDSs (Giustolisi et al., 2019). The decomposition of WDS is also of interest for solving hydraulic equations or optimal design (Deuerlein, 2008). Zheng et al. (2013) decomposed looped WDS into multiple shortest distance trees and then optimized that structure and subsequently included these results in the optimization of the entire looped network. Ciaponi et al. (2017) also showed the importance of minimum water path criteria and, therefore, the spanning tree of a looped WDS for optimal design. However, while the complex nature of individual WDSs has already been explored, literature is still lacking on an investigation of CNA of Pareto‐optimal networks. Further, the potential of CNA as an optimization procedure itself has not been addressed in literature. The reason is that for a CNA of WDS, usually, the topology (network layout) is known and kept constant; therefore, the topology‐based graph metrics of the Pareto‐optimal network are not changing for different solutions. Nevertheless, when looking at a multiobjective pipe design procedure, for the set of trade‐off (Pareto) solutions the diameters and, therefore, the hydraulic features of optimal networks are changing. These features can be included in the graph analysis as (flow) directions, different weights (e.g., flow, head loss, or capacity), or as aggregation criteria.

The optimal design of real WDSs is a many‐objective problem, which is usually (over)simplified (Fu et al., 2013). However, the complexity of including multiple objectives is out of the scope of this work. The aim of this work is to explore network characteristics of optimal networks. Therefore, different real WDSs are optimized with a two‐objective methodology considering costs and resilience (Wang et al., 2017). Based on an analysis of a multitude of optimal WDSs, network characteristics of optimal solutions are identified, and the applicability of graph measures is investigated. Based on the systematic investigations, a CNA design approach is developed, which is capable to improve computation efficiency of evolutionary algorithms for large networks. The new CNA design approach is tested on three real case studies and then applied to a very large WDS with 150,000 pipes.

## 2 Methodology

**Figure 1:**
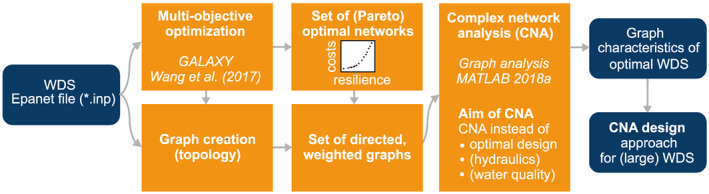
Workflow of this study.

The workflow of the proposed methodology is outlined in Figure 1. For the data interface, the WDS is represented as an Epanet2 input file (Rossman, 2000). The WDS is then converted to a Matlab graph object (MathWorks, 2018), which is the basis for further CNA. Sets of optimal WDS solutions are subsequently created and used to (1) identify characteristics of optimal solutions, (2) investigate systematically the applicability of CNA for optimization, and (3) develop and compare the outcome of the CNA design with the solutions based on evolutionary optimization.

### 2.1 Multiobjective Optimization

A WDS should provide potable water of sufficient quantity and quality. As these infrastructures are expensive to build and maintain, they have to be economically viable. However, additional performance metrics, such as reliability and leakage, are also important for these systems requiring a multiobjective approach for the WDS design problem (Cunha & Marques, 2020; Johns et al., 2019). Those types of problems are difficult to solve, especially for large complex networks (Wang et al., 2017). For a detailed introduction to WDS multiobjective optimization the reader is referred to recent review literature (Maier et al., 2019; Mala‐Jetmarova et al., 2017; Wang et al., 2017).

For the optimization work in this study, the state‐of‐the‐art methodology GALAXY (Genetically Adaptive Leaping Algorithm for approXimation and diversitY) is used, based on multiobjective evolutionary algorithms (Wang et al., 2017). The two conflicting objectives, that is, minimizing costs and maximizing resilience, are used for optimization. The total cost is calculated based on the unit pipe costs as a function of the discrete pipe diameters and pipe lengths. The network resilience *I*_*n*_ according to Prasad and Park (2004) is used (see Equation 1). Therein, the number of nodes #*N*, the number of sources #*S*, and the number of pumps #*P* with power supplied by pumps *P*_*i*_ are used. For each node *j* with the demand *Q*_*j*_ the surplus power as head *H*_*j*_ above the minimum required head *H*_min_ (in this study 30 m) is used, and for each source node *k* the discharge *Q*_*k*_ and head *H*_*k*_ is used:

(1) $$I_{n}=\frac{\sum_{j=1}^{\#N}C_{j}\cdot Q_{j}\cdot \left(H_{j}-H_{\min}\right)}{\left[\sum_{k=1}^{\#S}Q_{k}\cdot H_{k}+\sum_{i=1}^{\#P}P_{i}/\gamma \right]-\sum_{j=1}^{\#N}Q_{j}\cdot H_{\min}}$$

with the uniformity *C*_*j*_ of a node *j* to which *npj* pipes are connected with the diameters *DN*_*i*_,

(2) $$C_{j}=\frac{\sum_{i=1}^{\mathrm{npj}}{\mathrm{DN}}_{i}}{\mathrm{npj}\cdot \max\left({\mathrm{DN}}_{i}\right)}$$

For the optimization procedure, the hybrid replacement strategy implemented in GALAXY is used. A standard replacement strategy, as implemented in NSGA‐II (Deb et al., 2002), is applied if the number of top ranked individuals is not greater than the population size. If it exceeds the population size, an *ε* replacement strategy is applied, and, therefore, only the *ε*‐nondominated solutions are selected for the next generation (Wang et al., 2017). Parameters for the optimization process are described in more detail in the case study section (section 3).

### 2.2 CNA

#### 2.2.1 Graph Representation of WDNs

A WDN can be described with a link node representation of physical components (Yazdani & Jeffrey, 2011). Such a network can be represented as a mathematical graph *G*, which consists of a set *N* of #*N* graph nodes that are interconnected via a set *E* of #*E* edges (pipes, valves, etc.). For a WDN the set of demand nodes *D* are the sinks and are a subset of the vertices *N* (*D* ⊆ *N*). For a WDN, there has to be at least one source node with a fixed head, but there can also be a set of multiple sources *S* (*S* ⊆ *N*).

The adjacency matrix *A* (#*N* × #*N*) of *G* describes the physical connections between the nodes *N*. The element *a*_*ij*_ in *A* is either 1 if there is a connection between nodes *i* and *j* or 0 otherwise. The graph *G* can be directed (with a given flow direction) or undirected. For further analysis, for each edge a weight can be assigned. A graph is unweighted if all weights have the same value (usually 1). In a weighted graph, each edge *k* has a specific weight value. By using different terms for weights (*w*_*k*_), investigations with different objectives can be made with the same graph metrics. For example, the weights can represent friction losses; therewith pressure‐related investigations can be made. On the other hand, the weights can represent travel time; therewith water age or water quality‐related analysis could be performed.

In this work, an interface was implemented for creating Matlab graph objects for WDSs from Epanet2 input files. The interface also provides simulated parameters (flows, head losses, flow directions, etc.) calculated with the EPANET Programmer's Toolkit. The information is stored as additional graph data, which can be used for graph weights, attributes, or directions. With these weighted graphs of the WDSs, different kinds of graph analysis can be performed.

#### 2.2.2 Weights of the Edges

Yazdani and Jeffrey (2012a) and Meng et al. (2018) used unweighted and undirected graphs to identify topological attributes for network resilience analysis. However, they also outlined that proper weights can be useful for CNA. Herrera et al. (2016) investigated the resilience of WDSs and proposed to use weights as a surrogate for flow resistance, and they suggested the term pipe length divided by pipe diameter for resilience assessment. Yazdani and Jeffrey (2012b) suggested using the physical capacity of a pipe as pipe length times the squared pipe diameter as a weight for vulnerability analysis, which is not the focus of this study. Simone et al. (2019) used the pipes' connectivity, hydraulic resistance, and pipe length to move network domain analysis closer to expected hydraulic behavior.

As the trade‐off between cost and resilience is the main concern of this study, we use a different way of weighting method for edges. First, it is investigated to what extent analysis with appropriate weights can be used for pressure estimations. Therefore, expressions of friction losses are used as weights. The full description (implemented in hydraulic solvers) and subsequent different simplifications are investigated until the weighting only relies on structural properties without the need for the results from hydraulic simulations. That provides a systematic analysis of the impacts of different simplifications. Further, it is investigated if the pressure‐related analysis could be performed with CNA and what the inaccuracies along with it are. Finally, it is investigated to what extent the topological structure can be described using CNA with different weights.

In Epanet2 the Darcy‐Weisbach formula (in the following in SI units) can be used for calculation of head losses (*h*_*L*,*k*_) in each pipe *k* with the diameter *DN*_*k*_, the length *L*_*k*_, and the pipe flow *Q*_*k*_ (Rossman, 2000):

(3) $$h_{L,k}=A_{k}\cdot Q_{k}^{2}\;\left(m\right)$$

with

(4) $$A_{k}=\frac{0.0252}{0.3048}\cdot f\left(\varepsilon_{k},{\mathrm{DN}}_{k},Q_{k}\right)\cdot \frac{L_{k}}{{\mathrm{DN}}_{k}^{5}}$$

and *ε*_*k*_ being the Darcy‐Weisbach roughness coefficient. The friction factor *f* depends on the actual flow regime in the pipe. The flow regime can be expressed with the Reynolds number *Re*_*k*_ for each pipe *k*:

(5) Rek=DNk·vkν−

with the flow velocity *v*_*k*_ in each pipe *k* and *ν* as the kinematic viscosity (*ν* = 1.308·10^−6^ m/s^2^ for water with a temperature of 10°C, assumed constant). In Epanet2 for laminar flows (*Re* < 2,000) the Hagen‐Poiseuille formula is used for *f*. For fully turbulent flows (*Re* = 4,000) the Swamee and Jain approximation to the Colebrook‐White equation is used (Rossman, 2000):

(6) f=12·log10εk3.7·DNk+5.74Rek0.92

In the transition phase from laminar to turbulent flow (2,000 ≤ *Re* ≤ 4,000), a cubic interpolation of the Moody diagram is used (Rossman, 2000). Note that minor losses are neglected in this study. As the first weighting function *w*1_*k*_ the full implementation of the friction losses *h*_*L*,*k*_ is used:

(7) $$w1_{k}=h_{L,k}$$

In the second step, the parameters in the friction factor *f* are assumed constant (e.g., flow velocity *v*_*k*_ = 1.0 m/s, *ε*_*k*_ = 0.4 mm) resulting in a coefficient *c*_2_ (see further details on coefficients *c*_*i*_ in section 2.3). For the performed graph analysis, the absolute values of *c*_*i*_ are not relevant because it does not change the rankings of paths based on their lengths. Nevertheless, the actual path length is required for head loss estimations, because it converts the path lengths to friction losses. With the assumed simplifications, in *A*_*k*_ (Equation 4) the term $L_{k}/{\mathrm{DN}}_{k}^{5}$ remains. Using that term in Equation 3 results in *w*2_,*k*_:

(8) $$w2_{k}=c_{2}\cdot \frac{L_{k}}{{\mathrm{DN}}_{k}^{5}}\cdot Q_{k}^{2}$$

The pipe flow *Q*_*k*_ can be expressed according to the continuity equation with $Q_{k}=v_{k}\cdot {\mathrm{DN}}_{k}^{2}\cdot \pi /4$. When the flow velocity *v*_*k*_ in all pipes is assumed constant, it becomes a function of ${\mathrm{DN}}_{k}^{2}$. If we use this expression of *Q*_*k*_ in Equation 8, the following formulation is derived as the third weight:

(9) $$w3_{k}=c_{3}\cdot \frac{L_{k}}{{\mathrm{DN}}_{k}}$$

As a further simplification, the diameter *DN*_*k*_ is neglected (assumed the same value for all pipes), resulting in the Euclidian distance *L*_*k*_:

(10) $$w4_{k}=c_{4}\cdot L_{k}$$

#### 2.2.3 Graph Metrics

WDNs usually have a planar structure, which means that there are no edge intersections. In practice, such intersections might still happen when smaller pipes are not directly connected to transmission pipes and, therefore, cross each other without being connected. Nevertheless, a planar structure is assumed for simplicity. To characterize the analyzed WDNs, the *average node degree nd* (Newman, 2010) is used:

(11) $$\mathrm{nd}=\frac{2\cdot \#E}{\#N}$$

Further, the *meshedness coefficient r*_*m*_ (Buhl et al., 2006) indicates the actual number of independent loops in a WDN in relation to the maximum number of loops. The number of loops for a single‐sourced WDN can be computed with #*E* − #*N* + 1 (for multiple sources with #*E* − #*N*) and the maximum number of loops with 2#*N* − 5 which results in

(12) $$r_{m}=\frac{\#E-\#N+1}{2\#N-5}$$

The *shortest path length σ*_*i*,*j*_ is a path between nodes *i* and *j*, where the length is minimal. The length in this context is the sum of all weights of edges in the shortest path that connects nodes *i* and *j*. For determining the shortest path length, the different weights from section 2.2.2 and the Dijkstra (1959) algorithm are used. As a measure of the importance of edges in a WDN, the *edge betweenness centrality* (EBC) can be used. The EBC(*k*) values count how often an edge *k* is a part of the shortest paths between all node pairs *i* and *j*, normalized to the total number of the shortest paths #*σ* in the interval [0 1] (Girvan & Newman, 2002):

(13) $$\mathrm{EBC}\left(k\right)=\sum_{i\neq j\in N}\frac{\sigma_{i,j}\left(k\right)}{\#\sigma }\;\left[0\;1\right]$$

The graphs of WDNs have some specific characteristics; for example, every node has to be connected to at least one source node. Therefore, at least one path (i.e., the shortest path) also exists. In this work, a *source edge betweenness centrality* (EBC*) is used, which counts the number of the shortest path connections between a source node *i* and every demand (sink) node *j* (without normalization to #*σ*):

(14) $${\mathrm{EBC}}^{*}\left(k\right)=\sum_{i,j\in D}\sigma_{i,j}\left(k\right)\;\left[0\#N\right]$$

As the second modification, the *demand edge betweenness centrality* (EBC^*Q*^) is introduced which instead increases the count by 1 if an edge *k* is a part of the shortest path, and adds the demand of demand node *j* to the edge count:

(15) $${\mathrm{EBC}}^{Q}\left(k\right)=\sum_{i,j\in D}\sigma_{i,j}\left(k\right)\cdot Q_{j}\;\left[0\;\sum_{j\in D}Q_{j}\right]$$

**Figure 2:**
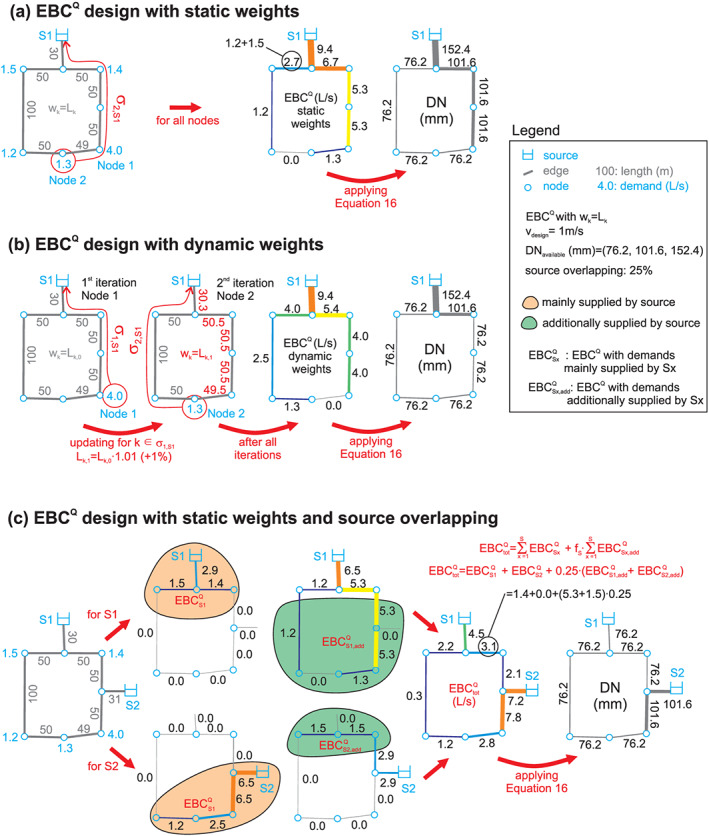
Illustrative examples for (a) EBC^*Q*^ design with static weights; (b) EBC^*Q*^ design with dynamic weights; and (c) EBC^*Q*^ design with static weights and source overlapping.

The values EBC^*Q*^ for each pipe *k* correspond, therefore, to water flow (denoted *Q*_EBC,*k*_) indicating how much of the total demand this pipe contributes to. An example how EBC^*Q*^ is determined can be found in Figure 2a (see also section 2.3).

The weights defined in section 2.2.2 are subsequently used to investigate systematically the topological structure of Pareto‐optimal WDNs with the different edge betweenness measures. The weights for estimating different measures can also be modified iteratively in the course of analysis, which is referred to as dynamic weights (see also Figure 2b). A demand parcel routed through the network would increase friction losses along that path. The next parcel would, therefore, tend to use an alternative path (similar to achieving the loop energy balance for hydraulic simulations). This hydraulics‐inspired principle of avoiding paths which already have a high *Q*_EBC,*k*_ can be used with CNA by increasing the weights of the edges in an identified shortest path to allow the next demand parcel to be routed.

When, for example, the pipe length (Figure 2b: *L*_*k*,0_) is used as a weight for routing the demand of Node 1 (Figure 2b) to the source (*σ*_1,*S*1_), the weights along that shortest path are increased for the next iteration (Figure 2b: *L*_*k*,1_), forcing alternative flow paths for the next demand parcel to be routed and resulting in lower *Q*_EBC,*k*_ values. This can be useful for avoiding very high EBC values and, therefore, utilize alternative flow paths in the networks.

### 2.3 Optimal Network Design Based on CNA

First, the network patterns of the obtained optimal solutions are investigated with EBC*. In order to obtain the maximum accuracy for determining the network patterns in optimal WDNs, *w*1_*k*_ = *h*_*L*,*k*_ is used (Equation 7) and compared with other simpler functions (Equations 8–11). Therefore, the question can be answered, if the results for EBC* can provide valuable information before any design procedure is applied.

In the second step, it is investigated to what extent the obtained results for the shortest path lengths with different weights can be used as head loss surrogates. For that, the nodal (graph) heads g*H*_*j*_ are not based on network hydraulics obtained by Epanet2 but estimated using the head loss *σ*_*i*,*j*_ from the source *i* to each node *j*. This is calculated by subtracting the head loss *σ*_*i*,*j*_ along the flow path from the source head *H*_*i*_ (g*H*_*j*_ = *H*_*i*_ − *σ*_*i*,*j*_). In the following, these head estimations with CNA are denoted as “graph heads” in contrast to the simulated heads computed by Epanet2.

In order to use the graph analysis with *w*4_*k*_ (Equation 10) for such pressure analysis, the coefficient *c*_4_ can be interpreted as friction slope (m/km). Based on the analysis of optimal WDS, the hydraulic gradient is assumed to be 20 m/km for the peak load case resulting in *c*_4_ = 1/50. When assuming an average pipe diameter of 0.1 m, *c*_3_ (Equation 9) can be estimated with 1/500. The coefficient *c*_2_ (Equation 8) is estimated from Equation 1 with an assumed constant flow velocity *v*_*k*_ = 1.0 m/s and the friction losses for all pipes with *ε*_*k*_ = 0.4 mm with *c*_2_ = 1/371. The values of *c*_*i*_ cannot be generalized and used for other cases unless the level of detail and technical design principals are comparable (e.g., the average pipe diameter is about 0.1 m).

In the last step, EBC^*Q*^(*k*) is used for each pipe *k* for the CNA design procedure. For that, the same set of discrete, commercially available diameter classes *DN*_available_ as in the multiobjective design procedure is used. With an assumed design velocity *v*_design_ the required diameter *DN*_*k*_ is determined according to the continuity equation and based on EBC^*Q*^(*k*) as next larger available diameter:

(16) $${\mathrm{DN}}_{k}=\left\lceil \left(\sqrt{\frac{4}{\pi }\cdot \frac{{\mathrm{EBC}}^{Q}\left(k\right)}{v_{\text{design}}}}\;\right\rceil \right)\in {\mathrm{DN}}_{\text{available}}$$

Regarding the design velocity *v*_design_ there is no fixed technical rule, but it is varied between 0.5 and 2.5 m/s (Walski et al., 2003) in order to cover a broad range of technically reasonable solutions. In one design iteration, the same *v*_design_ is used for all diameter classes. The design procedure is, therefore, only velocity based, and pressure constraints are not considered during the design process. Only after the design, the pressure constraints are checked with Epanet2, and the solutions which do not fulfill the pressure criteria are excluded. Note that for the CNA design procedure no hydraulic simulations are required, only for the final solutions the pressure constraints are checked with one simulation per design solution. To validate the obtained results, the solutions based on the CNA design are subsequently compared with the optimal solutions obtained with GALAXY.

In the illustrative example in Figure 2a, the EBC^*Q*^ based design with weights *L*_*k*_ is shown. For comparison in Figure 2b the design based on dynamic weights is shown. In the dynamic weight example, the shortest path from Node 1 to the source is determined first (*σ*_1,*S*1_) with weights *L*_*k*,0_. For the edges being part of *σ*_1,*S*1_, the pipe lengths are updated before the next iteration to *L*_*k*,1_. For Node 2, now *σ*_2,*S*1_ is determined with the updated weights *L*_*k*,1_. In contrast to *σ*_2,*S*1_ with static weights (taking the right branch), *σ*_2,*S*1_ for dynamic weights takes another route (left branch). When comparing the determined diameters of static and dynamic weights, one can see that with dynamic weights, solutions which better utilize redundant flow paths can be found. Note that the solution based on dynamic weights depends also on the order of demands processed, meaning using Node 2 in the first iteration would result in the same EBC^*Q*^ as for the static weights. The larger a network, the less important is this issue, but it could also be overcome by, for example, dividing a large demand in smaller packages. However, this solution requires more iterations. Another solution would be limiting the maximum lengthening during an iteration to a certain factor. For simplicity in the illustrative example a fixed lengthening factor of 1% of the previous length is used.

For using dynamic weights for the four real‐world case studies, after each demand routing through the network, that path length is increased by 1 + *Q*(L/s)^2^ up to a maximum increase of 1% per iteration. Or in other words, for *Q* = 0.1 L/s, the lengthening is determined with 1 + *Q*(L/s)^2^, and for *Q* ≥ 0.1 L/s a lengthening of 1% is used. The principal idea of using a path increase related to *Q* ^2^ is based on the quadratic approximation for pipe friction derived from the Darcy‐Weisbach equation (see Equations 3–6 and also weight *w*2_*k*_).

For *S* different sources, EBC* and EBC^*Q*^ have to be estimated for each of them separately resulting in *S* different values for *Q*_EBC,*k*_. Based on its location, each source mainly supplies a certain area (see also example in Figure 2c: ${\mathrm{EBC}}_{S1}^{Q}$ and ${\mathrm{EBC}}_{S2}^{Q}$). In addition, it can also contribute to areas farther away from it (see also Figure 2c: ${\mathrm{EBC}}_{S1,\mathrm{add}}^{Q}$ and ${\mathrm{EBC}}_{S2,\mathrm{add}}^{Q}$). To obtain a single value for each pipe *k* (see also ${\mathrm{EBC}}_{\mathrm{tot}}^{Q}$ in Figure 2), the different values can be again aggregated. This can be achieved, for example, by summing up the determined demands for each demand area or by applying a spatial strategy, for example, “overlapping strategy” (*f*_*S*_) of the supply areas (see Figure 2c: *f*_*S*_ = 25% overlapping of mainly supplied area and additionally supplied area). By applying this procedure for more than two sources, also different combinations of a spatial overlapping strategy can be considered.

## 3 Case Studies

**Figure 3:**
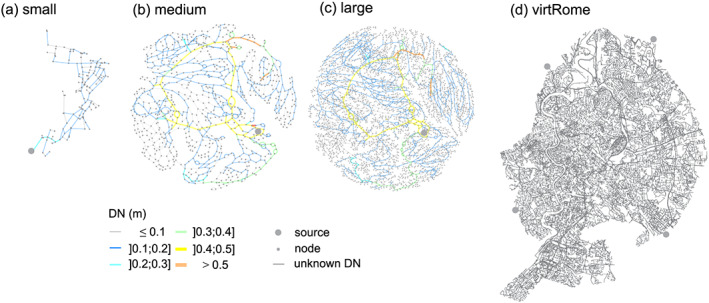
Graph drawing for the four case studies. (a) Small, (b) medium, (c) large, and (d) virtRome.

In this work, four case studies of different sizes are investigated, as shown in Figure 3. The proposed method is applied first to a small real network (242 nodes and 268 edges) with a length of 14 km (Figure 3a). Subsequently, the procedure is applied to a large real case with a length of 211 km to show its efficiency (Figure 3b). In addition, a skeletonized model with a length of 188 km for that large case is used, denoted as the medium case (Figure 3c). For the skeletonization process the WaterGEMS V8i is used by applying branch collapsing, series pipe merging, and smart pipe removal (Bentley Systems, 2006). Also, it is worth noting that the complexity of case studies considered in this study derives from the search space size which increases exponentially as the number of pipes rises. This is used to verify the effectiveness and efficiency of the proposed CNA on large real‐world design problems.

Due to security reasons, the real layouts of the medium and large case studies are not shown here. Instead of showing the graph in the Euclidean space, a graph drawing by force‐directed placement (Fruchterman & Reingold, 1991) is used, which aims to make edge length uniform and at the same time minimizes edge crossings (Figures 3b and 3c).

The skeletonized medium model has only one fourth of the nodes and one third of the edges of the large model (see Table 1). Nevertheless, the main hydraulic structure is preserved (see the main supply loop from the source with a diameter of 0.5 m). The average node degree (*nd*), as well as the meshedness coefficient (*r*_*m*_) for the medium case study, are clearly higher, as in the skeletonization process the branched structure is removed. This can also be seen in the differences in the node degree (*nd*) increasing from 2.26 to 3.06. Further, the average pipe length in the medium case is higher than those in the other two cases because of the merging of pipes in series. As a very large case study, a semivirtual case study of the city of Rome (virtRome) with more than 150,000 pipes is generated. The generation approach uses real open street map data and utilized the strong spatial correlation of water networks with street works (Mair et al., 2017) to create a possible supply network configuration (Sitzenfrei, 2016). To show the topographical characteristics of the different case studies, the 5% and 95% percentile (*P*_5_, *P*_95_) of the node elevations are also shown in Table 1. The small case has compared to its spatial extension, the largest height differences, while for the other cases, the topography of the WDS is relatively flat.

**Table 1:** Properties of the Case Studies

	#*N*	#*E*	*nd*	*r*_*m*_	Total length (km)	Average length (m)	*Q*_design_ (L/s)	Elevation (m) *P*_5_–*P*_95_
Small	242	268	2.215	0.0564	14	53.96	22.5	591.5–634.5
Medium	874	1,337	3.060	0.2262	188	140.70	1,132	565.3–590.9
Large	3,558	4,021	2.260	0.0653	211	52.68	1,132	565.2–603.7
virtRome	150,630	157,044	2.085	0.0213	3,832	24.40	6,000	17.5–67.5

For the optimization of the small, medium and large cases using GALAXY, a population size of 100 was used with 10,000 generations, resulting in 1 million function evaluations. For the large network, an additional scenario was run with 100,000 generations. Nine available discrete diameters ranging from 76.2 to 304.8 mm with unit costs from 8 to 150 € per meter length were used in the calculations for the small case study, and 15 diameters ranging from 76.2 mm to 914.4 mm with unit costs from 8 to 1,200 € per meter length were used for the medium and large case. For virtRome, an optimization with GALAXY was too computationally intensive. Therefore, only the CNA‐based design approach was applied.

## 4 Results and Discussions

### 4.1 Characteristics of Optimal Networks

**Figure 4:**
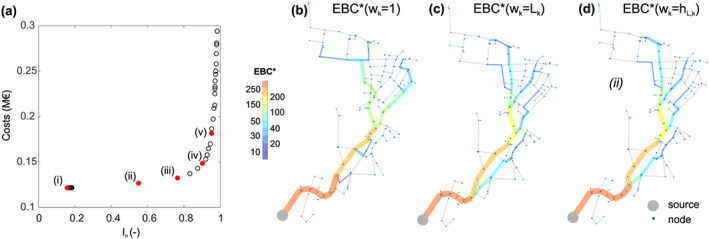
(a) Pareto front of 100 optimal (hydraulically feasible) solutions for the small case and five selected Solutions (*i*)–(*v*); (b) EBC* for that network with equal weights (*w*_*k*_ = 1), (c) Euclidian distance as weights (*w*_*k*_ = *L*_*k*_); and (d) EBC* of the optimized Network Solution (*ii*) with *w*_*k*_ = *h*_*L*,*k*_.

In Figure 4a, the Pareto front of the optimal solutions for the small case is shown (in total 100 solutions). *I*_*n*_ values close to 1 indicate the highest pressure reserve and therefore in this context the highest resilience values but also the highest pipe construction costs. In Figure 4b, EBC* is shown for the small case with an equally weighted graph (*w*_*k*_ = 1). In Figure 4c, EBC* is shown for weights according to the Euclidian distance (*w*_*k*_ = *L*_*k*_). For all Pareto‐optimal solutions (OPT), these two graphs (*w*_*k*_ = 1 and *w*_*k*_ = *L*_*k*_) look the same, because changes in diameters do not change the weights and therefore the results. If the friction losses are used as weighting (e.g., *w*_*k*_ = *h*_*L*,*k*_) for each OPT, a different EBC* can be determined. In Figure 4d EBC* for Solution (*ii*) (see Figure 4a with *I*_*n*_ = 0.55) is shown.

**Figure 5:**
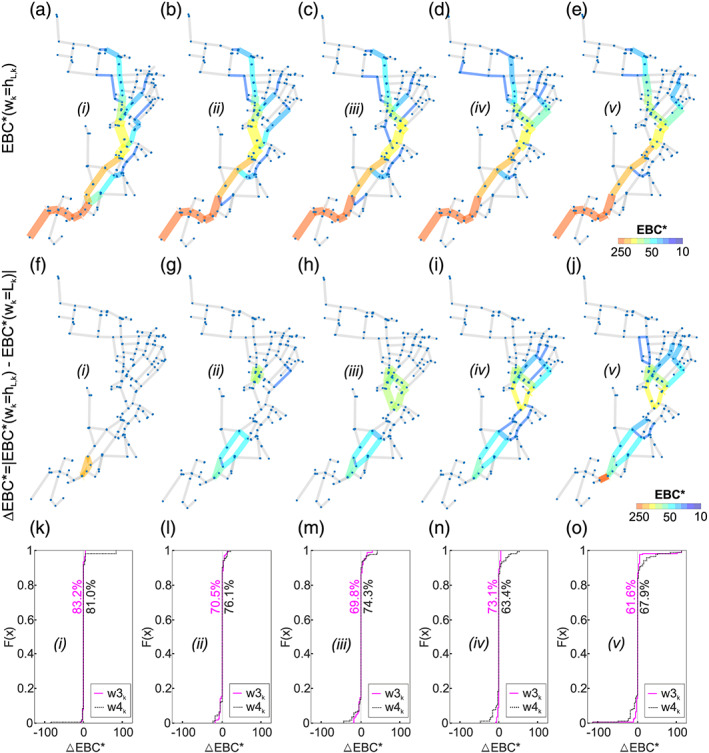
(a–e) EBC*(*w*_*k*_ = *h*_*L*,*k*_) for the five selected Optimal Solutions (*i*) to (*v*) for the small case; (f–j) absolute value of the difference between EBC*(*w*_*k*_ = *h*_*L*,*k*_) and the EBC*(*w*_*k*_ = *L*_*k*_) for Solutions (*i*) to (*v*); (k–o) CDF of the differences between EBC* (ΔEBC*) with different weights.

In Figures 5a to 5e a variety of the EBC* with *w*_*k*_ = *h*_*L*,*k*_ of the optimal layouts are shown. Solutions (*i*) to (*v*) in Figure 5 correspond with the marked solutions in Figure 4a. From visual comparisons of these solutions with Figure 4c (*w*_*k*_ = *L*_*k*_), Figures 5a to 5c follow the shortest Euclidean path lengths. In other words, low resilience solutions are driven by the shortest pipe lengths. The solutions in Figures 5d and 5e with higher resilience values differ from this shortest pipe length EBC*. To better point out this observation, in Figures 5f to 5j the absolute values of the differences between EBC*(*w*_*k*_ = *h*_*L*,*k*_) and the EBC*(*w*_*k*_ = *L*_*k*_) denoted ΔEBC* is shown. One can see that there are only marginal differences for Solution (*i*) and that the differences increase with increasing resilience values. It can also be observed that differences form loops with equal values. In Figures 5k to 5o the cumulative distribution functions (CDF) of ΔEBC* (as difference to EBC* determined with *w*_*k*_ = *h*_*L*,*k*_) are shown. The shown precentage indicate how much of the ΔEBC* values are 0 (no difference to EBC* with *w*_*k*_ = *h*_*L*,*k*_). For example, for ΔEBC* of *w*3_*k*_ as difference between EBC*(*w*_*k*_ = *h*_*L*,*k*_) and EBC* (*w*_*k*_ = *L*_*k*_/*DN*_*k*_), 83.2% of the obtained values are 0, meaning that 16.8% of the values are different.

These percentage values are decreasing with increasing *I*_*n*_. One can interpret that with increasing resilience values of the optimal solutions (according to the resilience measure used in this work), redundant capacities gain a more significant role in the diameter distribution. Therefore, the EBC* is more important for less resilient solutions. EBC* changes with high *I*_*n*_ values, and the shortest path criteria get less important for the design process.

The differences between EBC* values *w*_*k*_ = *L*_*k*_ and *w*_*k*_ = *L*_*k*_/*D*_*k*_ are insignificant. For this case study, it can be concluded that for low *I*_*n*_ EBC* using the *L*_*k*_ or *L*_*k*_ */D*_*k*_ as weights both can represent the near‐optimal layouts. The advantage of using *L*_*k*_ as weight is that no diameter information (and therefore no additional design procedure) is required to investigate a network.

### 4.2 CNA for Optimization

After investigating the importance of the shortest paths in optimal solutions, one can also hypothesize that the occurring friction losses can be assessed in a simplified way based on CNA. Obviously, if the friction losses *h*_*L*,*k*_ are used as weights, the distance of the shortest path from the source to a demand node represents the occurring friction losses. Therefore, when subtracting the occurring friction losses from the source head to a demand node, the pressure head in that demand node can be determined. To use *h*_*L*,*k*_ as weights, hydraulic simulations in advance are necessary. Note that the aim is to investigate to what extent CNA can be used as a surrogate for pressure analysis without hydraulic simulations. Therefore, the different simplifications for weights (Equations 7–11) are systematically investigated, and the impact on pressure estimations are quantified.

**Figure 6:**
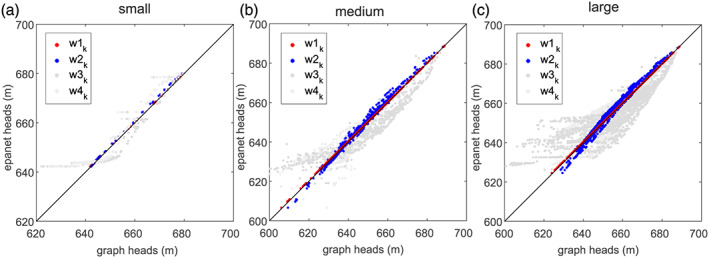
Comparisons of heads from graph analysis (*x* axis) and Epanet2 simulations (*y* axis) for different weights (*w*_*ik*_) and different cases (a) small, (b) medium, and (c) large.

In Figure 6 the graph heads in the nodes are estimated with CNA (*x* axis) and different weights (*w*_*i*,*k*_) and compared with heads determined with the Epanet2 (*y* axis). In Figures 6a to 6c, both heads from solutions of the small, medium, and large case are shown. One dot represents one node in a case, and the color of a node indicates which weight is used. Dots on or close to the black lines indicate that the graph heads are in good agreement with the Epanet2 heads. Using *w*1_*k*_ as weights results in good agreement and validates the methodology. From a visual comparison, using *w*2_*k*_ as weights (neglecting the Swamee and Jain approximation to the Colebrook‐White equation) also results in quite good agreement. However, for that weight, the pipe flow *Q*_*k*_ is required, which inevitably requires hydraulic simulations. For the weights *w*3_*k*_ and *w*4_*k*_ the differences in the two types of heads increase with underestimations and overestimations of the heads. Although the general trend is reproduced by the graph heads, the head differences between graph heads and heads determined with Epanet at a single node reach up to 30 m. For minimum pressure analysis, as here used for resilience estimation, these deviations are too high. Therefore, CNA is not suitable as pressure surrogate for such detailed analysis.

### 4.3 CNA Design Approach

**Figure 7:**
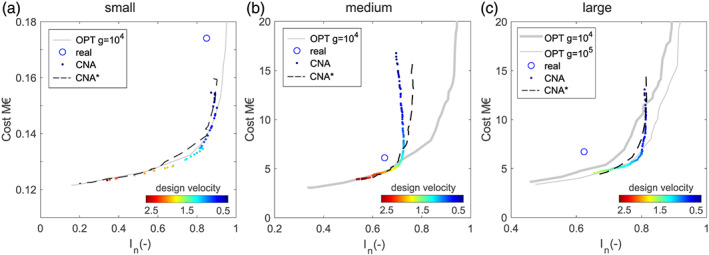
(a–c) Pareto fronts of optimal solutions (OPT) with *g* as the number of generations compared to CNA and CNA* design for the different cases.

In Figure 7, the results of this CNA design approach (dots) are shown for three different cases and compared with the OPT obtained with GALAXY. In addition, the real design is shown (the circle marker) to facilitate the comparison. In the CNA design based on EBC^*Q*^ (with *w*_*k*_ = *L*_*k*_), the design velocities vary from 0.5 to 2.5 m/s using 0.01 m/s steps, which results in 201 options. After the EBC^*Q*^ design, the costs are determined, and *I*_*n*_ are calculated using Epanet2 results. Solutions that do not fulfill the pressure constraints of 30 m are identified and removed. For the small and medium cases, all designs fulfill the pressure criteria; for the large case 113 designs fulfill those pressure criteria.

In Figure 7a the optimal solutions for the small case are shown in comparison with the 201 solutions obtained with CNA design. The colors of the CNA solutions indicate the used design velocities. The optimal solutions obtained with GALAXY cover a broader range of solutions in terms of costs and resilience. However, out of the 201 solutions determined with EBC^*Q*^, 189 provide more resilient solutions with lower costs compared to those by GALXY. With the discrete diameter classes, from the 201 solutions, only 48 unique solutions are obtained (e.g., the solution for 0.750 m/s is the same as for 0.751 m/s). Only the determined high resilient solutions (*I*_*n*_ > 0.9) are more expensive than those obtained with GALAXY with 1 million function evaluations (decision variable 268, population size 100, and generations [*g*] 10,000).

For the medium case, in Figure 7b some of the low resilience solutions are also found with lower costs (*I*_*n*_ from 0.54 to 0.71) compared with the optimization procedure (decision variables 1,337, population size 100, and generations [*g*] 10,000). Due to the complexity of the case study, all of the obtained 201 solutions are unique. For *I*_*n*_ values above 0.71, the *I*_*n*_ values do not increase anymore (although the costs do) because for the high resilient solutions the pipe redundancies become more important. In other words, efficient solutions do not concentrate the flow to the pipes with high EBC^*Q*^ values anymore. Nevertheless, very promising results are obtained with the proposed CNA design approach for low *I*_*n*_ values (=0.71) and design velocities above 1.25 m/s. To obtain the dotted black line (CNA*) for determining EBC^*Q*^, dynamic weights are used; that is, after each demand routing through the network, that path length is increased by 1 + *Q*(L/s)^2^ up to a maximum increase of 1% per iteration. Therewith, for the medium case study also solutions with resilience values closer to the optimal one can be obtained. For the other case studies, there is less difference in CNA and CNA*.

In Figure 7c, the results for the large case are shown. The minor distribution structure results in the longest flow paths and the largest supply area. Therefore, 88 of the obtained 201 solutions do not fulfill the minimum pressure criteria of 30 m, but due to the complexity of that case study, all of the remaining 113 solutions are unique. Almost all solutions obtained with CNA design are more resilient with lower costs than those obtained by GALAXY (generations [*g*] 10,000). Therefore, another scenario (very computationally intensive) with 100,000 generations, resulting in 10 million function evaluations, for the large case is included. These 10 million function evaluations were not optimized in terms of runtime (Matlab code, single core evaluations) and took 8 weeks on a desktop computer (Intel® Core™ i5–6,500 CPU @ 3.2 GHz). Compared to that, the CNA design approach based on EBC^*Q*^ was also not optimized in terms of runtime (Matlab code, single core evaluations) and took in total 30.3 s. The results of the 10 million function evaluations are shown as a thin gray line in Figure 7c (OPT *g* = 10^5^). Even for these OPT, some of the CNA‐based designs outperform the optimized solutions. Nevertheless, for high resilient solutions, the approximation of the Pareto front is less efficient. But note that for the large case, that is, for *I*_*n*_ > 0.75, the travel time in the network (determined with demand patterns but without tank aging) is already longer than 7 days, which makes that solution less desirable from the water quality perspective. Further, when comparing to the real designs for the three cases (circular markers in Figure 7), higher resilience values can be achieved with less costs by CNA and CNA*. This also supports the argument that technically desirable resilience values can be achieved with the proposed CNA design approach.

**Figure 8:**
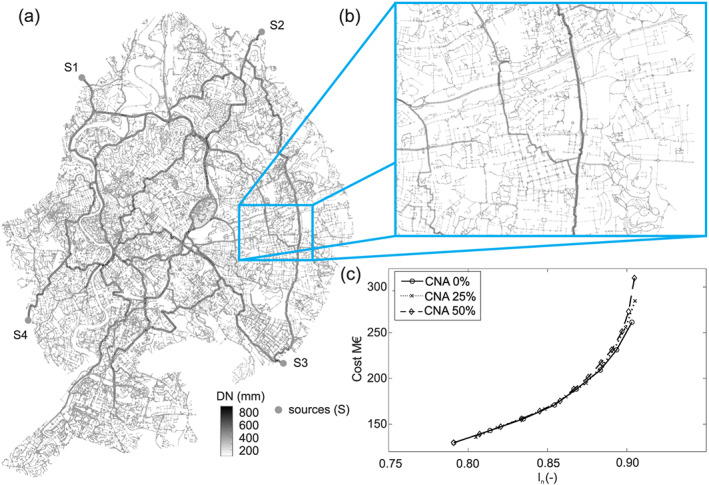
(a) Layout and diameters of one solution for the virtRome; (b) detail of the virtRome; and (c) solutions obtained with CNA and scenarios for partly redundant capacity (CNA 25% and CNA 50%).

In Figure 8, the results of applying the CNA design approach to the virtRome case is shown. The case has four sources. For each source, the supply area can be assigned with the shortest path analysis, and each supply area can be designed accordingly (CNA 0%). In addition, 25% and 50% overlapping of supply areas were considered (CNA 25% and CNA 50%), introducing an overcapacity in the system. The results of these (optimal) solutions are shown in Figure 8c.

In Table 2, the numerical performance (i.e., runtime) for obtaining the different solutions are summarized. The CNA design approach is split up in reading the Epanet2 input file and creating the graph object, determining EBC^*Q*^, running the 201 design scenarios, calculating costs and *I*_*n*_ with Epanet2, and checking the minimum pressure criteria. In total, it took 2.96 s to design 201 solutions and check the constraints for the small case. In contrast to that, it took 2:21 hr to do the optimization with GALAXY (a factor of 2.9 × 10^3^ in runtime). For the medium case, in total, it took 15.26 s for the CNA design and 37.5 hr for the optimization procedure (factor 8.8 × 10^3^ in runtime). Designing with CNA, the large network took 30.3 s, and with a population size of 100 and generation number of 10,000 it took 146 hr to obtain the optimal solutions (factor 1.7 × 10^4^ in runtime). For the scenario with 100,000 generations, the CNA design was approximately a factor 10^5^ faster.

**Table 2:** Numerical Performance of Approaches

	Create graph (s)	EBC^*Q*^ (s)	EBC^*Q*^ design #201 (s)	*I*_*n*_, costs (s)	#EBC^*Q*^ solutions (#)	GALAXY *p* = 100, *g* = 10^4^ (hr)	GALAXY *p* = 100, *g* = 10^5^ (weeks)
Small	0.68	0.21	0.09	1.98	201 (48)	2:21	—
Medium	2.01	1.13	0.10	11.97	201	37:36	—
Large	3.86	3.11	0.15	38.19	113	146:01	8
virtRome	146.7	4,175	3.36	1,075	13	—	—

The proposed CNA design approach based on EBC^*Q*^ only provides a smaller range of solutions compared to a traditional multiobjective optimization approach. Nevertheless, it achieves remarkable results for a specific resilience range, in which the optimal solutions are driven by the shortest path criteria, and redundancy does not play a crucial role. It is hypothesized that evolutionary algorithms like GALAXY will also identify these optimal solutions by the CNA design approach with sufficient population sizes and generations, but a tremendously higher computational burden would be imposed. Although this might be acceptable for small networks, the bigger the network to design, the more important it gets to identify solutions in a computationally efficient manner.

The proposed design method is especially of interest for large networks with a few thousand pipes or even more. Also, when applying the design procedure to smaller networks but in a great number (Guo & Englehardt, 2015; Möderl et al., 2011; Sitzenfrei et al., 2010) or in course of integrated studies, in which many different supply options based on uncertainty analysis are performed (Rauch et al., 2017; Sitzenfrei et al., 2013), such an approach can be of great help.

## 5 Summary and Conclusions

The aim of this study was to investigate the characteristics of optimal networks and show to what extent the CNA can be used to improve the optimization of WDSs. Therefore, three real case studies were optimized and subsequently investigated based on CNA. The obtained sets of OPT were subsequently examined to

1. identify network characteristics of optimal solutions;
2. investigate the applicability of CNA for optimization of WDSs with a systematic investigation of graph weights in combination with graph measures (the shortest paths analysis and the customized edge betweenness centrality EBC* and EBC^*Q*^);
3. validate whether the newly developed CNA design approach, which is based on the results from (1) and (2), is efficient in terms of computational time and applicable to large networks (i.e., more than 150,000 pipes).

In terms of characteristics of optimal networks, it is concluded that the less resilient solutions can be described better with the EBC* based on Euclidean distance as weights. With high resilience values, the shortest path criteria become less important for the design process. Therefore, it can be concluded that the EBC* using the Euclidian distances as weights represents the optimal layouts for a certain range of resilience values (based on the resilience definition used in this work). Based on a systematic investigation of pressure analysis in combination with CNA, it is determined that CNA is not a reliable surrogate for pressure‐based resilience estimations.

With the results obtained above, a CNA‐based design approach was developed, which was based on the customized edge betweenness centrality (EBC^*Q*^) for WDS analysis. The CNA design procedure does not require hydraulic simulations during the optimization process as the results need to be checked only afterward to confirm that the given pressure constraints are fulfilled. When compared to short execution times of the order of a few seconds required for the CNA design, the evolutionary optimization required hours or even weeks of computation time to obtain the results for the large networks (i.e., the CNA design is faster with a factor of up to 10^5^). However, the quality of the CNA design solutions is limited, when compared to the evolutionary algorithm results, although dynamic weights can be used to increase the covered resilience range.

The proposed approach can provide useful approximate results for tasks where a simple and fast design without evolutionary optimization is required or possible. In addition, for a practical engineering task, where a fast feasibility analysis, that is, without a wide range of solutions is needed, the proposed method could be implemented in engineering software. Different design velocities can be used for different diameter classes, which is beyond the scope of this work but will be the subject of future research. It can also be of interest to use the obtained results from the CNA design approach as the initial population of traditional optimization approaches (e.g., GALAXY) to facilitate a faster convergence to the Pareto front of optimal solutions.

The proposed CNA design approach could also be adapted to other pressure‐based resilience measures or network analysis‐based measures. This, however, requires further investigations. Furthermore, as already shown in Sitzenfrei et al. (2019), graph analysis with appropriate weights (in that case travel time) can be used as a surrogate measure for computationally efficient water quality analysis, enabling the inclusion of water quality in evolutionary optimization procedures with low additional computational efforts. A combination of the proposed CNA design approach with the CNA‐based water quality assessment will introduce another objective in this multiobjective approach in future work.

## Data Availability

The data of virtRome can be accessed via the data repository of the Unit of Environmental Engineering of the University of Innsbruck (https://www.uibk.ac.at/umwelttechnik/softwareanddatasets/).
